# Phenolic Profiles and Antioxidant Activity of Litchi (*Litchi Chinensis* Sonn.) Fruit Pericarp from Different Commercially Available Cultivars

**DOI:** 10.3390/molecules171214954

**Published:** 2012-12-17

**Authors:** Wu Li, Hong Liang, Ming-Wei Zhang, Rui-Fen Zhang, Yuan-Yuan Deng, Zhen-Cheng Wei, Yan Zhang, Xiao-Jun Tang

**Affiliations:** Key Laboratory of Functional Food Research, Ministry of Agriculture, Bio-Technical Research Institute, Guangdong Academy of Agricultural Sciences, Guangzhou 510610, China; E-Mails: leewuu@163.com (W.L.); lianghong_1025@163.com (H.L.); ruifenzhang@163.com (R.-F.Z.); yuanyuan_deng@yeah.net (Y.-Y.D.); zhencheng_wei@163.com (Z.-C.W.); zhang__yan_@126.com (Y.Z.); xjtang66@163.com (X.-J.T.)

**Keywords:** phenolics, anthocyanins, procyanidins, litchi fruit pericarp, antioxidant activity

## Abstract

Litchi fruit pericarp (LFP) contains significant amounts of phenolics which have been found to exhibit diverse biological activities. The purpose of this work was to determine the varietal differences in phenolic profiles and antioxidant activity of LFP from nine commercially available cultivars. The total phenolic and flavonoid contents ranged from 9.39 to 30.16 mg gallic acid equivalents/g fresh weight (FW) and from 7.12 to 23.46 mg catechin equivalents/g FW, respectively. The total anthocyanin contents ranged from 1.77 to 20.94 mg cyanidin-3-glucoside equivalents/100 g FW. Three anthocyanins, including cyanidin-3-rutinoside, cyanidin-3-glucoside, malvidin-3-glucoside, were detected, and cyanidin-3-rutinoside was the predominant constituent which contributes from 68.8% to 100% to total anthocyanins, The total procyanidin contents ranged from 4.35 to 11.82 mg epicatechin equivalents/g FW. Procyanidin B2, epicatechin, A-type procyanidin trimer, and procyanidin A2 were detected in all nine litchi varieties. The oxygen radical absorbance capacity activities and DPPH radical-scavenging activities ranged from 430.49 to 1752.30 μmol TE/100 g FW and from 4.70 to 11.82 mg/g (IC50), respectively. These results indicate that there are significant differences in phytochemical profiles and antioxidant activity among the tested varieties. Knowing the phenolic profiles and antioxidant activity of LFP of different varieties gives the insights into its potential application to promote health.

## 1. Introduction

Polyphenols are naturally occurring compounds widely found in the fruits, vegetables, cereals and other plants, which contribute to the color, flavor and defend a plant from ultraviolet rays or pathogens [1]. Accumulating epidemiological studies have demonstrated that increased consumption of fruits and vegetables can reduce risk of chronic diseases associated with oxidative stress, such as cardiovascular disease, cancer, diabetes and neurodegenerative diseases [2,3,4]. The protection of fruits and vegetables against these diseases has been attributed in part to their significant amounts of phenolics, which have potent antioxidant activity. Previous researches have demonstrated that plant polyphenols can reduce oxidative damage to biomolecules and regulate gene expression by modulating reactive oxygen species [5].

Litchi (*Litchi chinensis* Sonn.) is a tropical and subtropical fruit native to China, and now widely cultivated throughout the World. It is well received by consumers because of its delicious taste and possible health benefits, and its processing production has steadily increased in late decades. Litchi fruit pericarp (LFP) accounts for approximately 15% by weight of the whole fresh fruit and contains significant amounts of phenolics which are usually discarded as a waste in the process [6,7]. The phenolics of LFP have been confirmed to have antioxidant, anticancer [8], immunomodulatory [9] activities. LFP has been considered a new source of pharmaceuticals and food industry.

There are discrepancies in the phenolic profiles of LFP reported in previous studies. Prasad *et al.* [10] reported that the major flavonoid compounds extracted from LFP were epicatechin and epicatechin gallate. Zhao *et al.* [6] identified the major flavonoid chemicals extracted from LFP as procyanidin B2, procyanidin B4 and epicatechin. Liu *et al.* [11] found that the main oligomeric procyanidins isolated from LFP were epicatechin, procyanidin A2 and A-type procyanidin trimer. The phenolic content of LFP reported by Ruenroengklin *et al.* [12] is about 6-fold different from that reported by Prasad *et al.* [13]. It is not clear whether the differences in these studies are attributable to genotype or environmental conditions [7]. Moreover, little is known about the complete profiles of phenolic compounds and antioxidant activity of LFP of different varieties. The biological activity of the phytochemical extracts was associated with their composition and contents of activity ingredient. Therefore, it is important to determine the phenolic contents and characterize individual bioactive compounds of litchi varieties. The aims of this study thus were: (1) to determine phenolic profiles and antioxidant activity of LFP of nine commercially available varieties of litchi; (2) to compare the varietal differences of phytochemicals and antioxidant activity; and (3) to investigate the correlations among total phenolics, flavonoids, anthocyanins, *procyanidins* and antioxidant activity of the tested samples.

## 2. Results and Discussion

### 2.1. Contents of Total Phenolics, Flavonoids, Anthocyanins, and Procyanidins of the Pericarp of Litchi Varieties

The phenolic profiles of the nine litchi varieties are shown in Table 1. The total phenolic contents of the nine varieties ranged from 9.39 to 30.16 mg GAE/g FW, with an average of 16.27 mg GAE/g. The coefficient of variation (CV) of phenolic content in litchi varieties was 43.3%, indicating that there were significant genotype differences in phenolic contents among the litchi varieties. A 3.2-fold difference in phenolic content was found between the highest and lowest varieties, Heiye and Chanchutou (*p* < 0.05), respectively. 

The total flavonoid contents of the nine litchi varieties ranged from 7.12 to 23.46 mg CAE/g FW with an average of 12.82 mg CAE/g FW (Table 1). Like the total phenolic contents, there were significant differences in the total flavonoid contents among varieties. Heiye variety had the highest flavonoid content (23.46 mg CAE/g FW), with a 3.3-fold difference compared to the variety with the lowest flavonoid content (Jizui, *p* < 0.05). 

The total anthocyanin contents of the nine litchi varieties ranged from 1.77 to 20.94 mg CGE/100 g FW with an average of 8.73 mg CGE/100 g FW (Table 1). Huaizhi and Heiye varieties had higher anthocyanin contents (20.94 mg CGE/100 g and 18.60 mg CGE/100 g, respectively), 11.8-fold and 10.5-fold higher when compared to the variety with the lowest anthocyanin content (Feizixiao, *p* < 0.05). The total procyanidin contents of the nine litchi varieties ranged from 1.71 to 7.46 mg EPE/g FW with an average of 4.82 mg EPE/g FW (Table 1). A 4.4-fold difference in total procyanidin content was found between the highest and lowest varieties, Heiye and Jizui (*p* < 0.05), respectively.

The present results clearly showed that there was a great diversity in total phenolic, flavonoid, anthocyanin, and procyanidin contents. Previous studies also showed a difference in phenolic content inf LFP, as reported by Prasad *et al.* [13] with 16 mg/g dry weight in Baila variety and by Ruenroengklin *et al.* [12] with 100 mg/g dry weigh in Feizixiao variety. The phenolic levels of the Heiye, Huaizhi, Guiwei, Feizixiao and Dingxiang varieties in the present work were higher than the level of 13.90 mg/g reported by Duan *et al.* [14]. These differences may be attributed to different cultivars or the methodology used for phytochemical analyses. The total phenolic contents of tested litchi varieties were comparable to that of the grape seed with 1.4–22.3 mg GAE/g [15] and skin with 4.9–13.8 mg GAE/g [16]. Therefore, litchi fruit pericarp is a good source for phenolic compounds.

The anthocyanin contents of Heiye and Guiwei in the present study were consistent with the level (18.6 mg/100 g FW) reported by Duan *et al.* [14], but lower than the level (52.0 mg/100 g FW) reported by Rivera-Lopez *et al.* [17]. The total anthocyanin contents of tested litchi varieties were comparable to that of red raspberry (6.56–29.60 mg/100 g) [18] and strawberry (15.0–35.0 mg/100 g) [19]. The total procyanidin contents in most tested litchi varieties were higher than the level (2.8 mg/g FW) reported by Sarni-Manchado *et al.* [20], but lower than the level (8.2 mg/g FW) reported by Zhang *et al.* [21]. The procyanidin contents in all tested litchi varieties except Jizui were even higher than that in blueberry (3.32 mg/g FW) [22], which has been considered a high procyanidin-containing fruit. Therefore, the fruit pericarp of some litchi varieties can be used to extract anthocyanins and procyanidins for nutraceutical development or food colorants.

### 2.2. Anthocyanin Compositions of the Pericarp of Litchi Varieties

**Figure 1:**
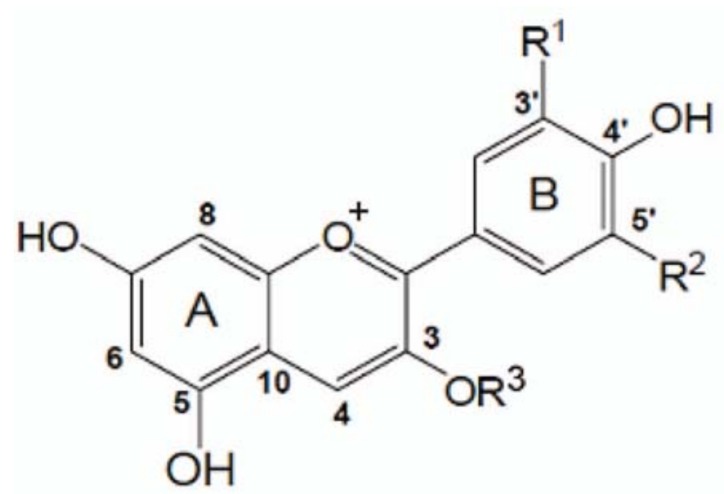
Chemical structures of three anthocyanins detected in nine litchi varieties: (1) cyanidin-3-glucoside, R^1^ = OH, R^2^ = H, R^3^ = glucose; (2) cyanidin-3-rutinoside, R^1^ = OH, R^2^ = H, R^3^ = rutinose; (3) malvidin-3-glucoside, R^1^ = R^2^ = OCH_3_, R^3^ = glucose.

Anthocyanin compositions of the nine litchi varieties tested are presented in Table 2. Three anthocyanins were detected in the nine varieties, including cyanidin-3-glucoside, cyanidin-3-rutinoside, and malvidin-3-glucoside. The chemical structures of these standard compounds are shown in Figure 1. Cyanidin-3-rutinoside, which was detected in all nine litchi varieties, was the predominant anthocyanin constituent and contributed 68.8% to 100% of the total anthocyanins. Its content ranged from 1.29 mg/100 g FW in Feizixiao to 19.11 mg/100 g FW in Huaizhi. Malvidin-3-glucoside was detected in all tested litchi varieties except Feizixiao and Changchutou. Its content ranged from 0 to 1.98 mg/100 g FW. Cyanidin-3-glucoside was detected in Heiye, Huaizhi and Changchutou. Its content ranged from 0.80 to 1.18 mg/100 g FW.

In this and the majority of previous studies, cyanidin-3-rutinoside was identified as the predominant anthocyanin in LFP [17,20]. The cyanidin-3-rutinoside contents of Heiye and Huaizhi varieties in the present study were consistent with the value (17 mg/100 g FW) reported by Duan *et al.* [23], and Zhang *et al.* [24], but were lower than the value reported by Sarni-Manchado *et al.* (42.9 mg/100 g FW) [20], and Martinez-Castellanos *et al.* (80.0 mg/100 g FW) [25]. Anthocyanin composition of LFP reported in previous studies was distinct. Sarni-Manchado *et al.* [20] reported that cyanidin-3-rutinoside and cyanidin glucoside were the major anthocyanins in pericarp tissues of Kwai Mi variety. While Lee and Wicker [26] and Rivera-Loapez *et al.* [17] found that the major anthocyanins in pericarp tissues of Brewster variety were cyanidin-3-glucoside, cyanidin-3-rutinoside and malvidin-3-glucoside. These differences of anthocyanin profiles in these studies may reflect cultivar differences. It was supported by this work that found significant differences in anthocyanin compositions and contents among tested litchi varieties. 

### 2.3. Procyanidin Compositions of the Pericarp of Litchi Varieties

**Figure 2:**
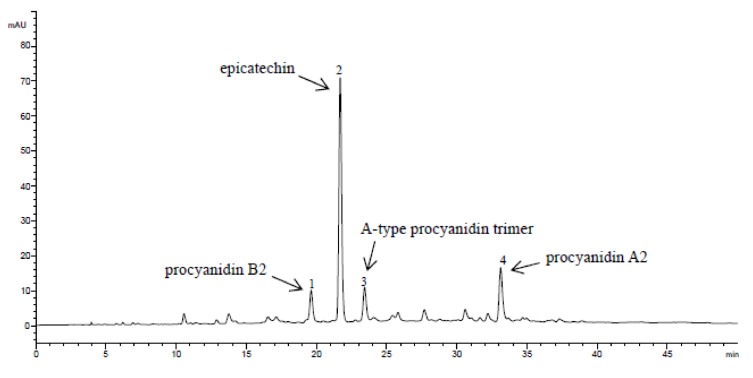
Typical HPLC chromatographic profile of procyanidins of litchi pericarp. 1: compound **1**; 2: compound **2**; 3: compound **3**; 4: compound **4**.

The main oligomeric procyanidins of tested litchi varieties were identified by HPLC/MS. Figure 2 shows the typical HPLC chromatogram of the procyanidins of the tested litchi varieties. Four major compounds were found in the pericarp of all the nine litchi varieties. Compound 1 exhibited molecular ion ([M−H]^−^) at *m/z* 576.5, in the respective MS^2^ spectrum, and three fragment ions were found at *m/z* 288.2, 424.4 and 514.4, respectively. According to previous references [6,11] about the procyanidins of litchi pericarp, it was identified to be procyanidin B2. Compound 2 exhibited a molecular ion ([M−H]^−^) at *m/z* 289.1, in the respective MS^2^ spectrum, and its fragment ions were found at *m/z* 245.1, 205.0 and 179.0. According to the previous references [6,11], it was identified as epicatechin. Compound **3** exhibited a signal of molecular ion ([M−H]^−^) at *m/z* 862.7, in the respective MS^2^ spectrum, and its fragment ions were found at *m/z* 288.4, 410.5, 450.5, 530.6, 558.6, 572.6, 692.6, 710.6, 736.6 respectively. It was identified as A-type procyanidin trimer as reported by Liu *et al.* [11]. Compound 4 exhibited molecular ion ([M−H]^−^) at *m/z* 574.5, and the fragment ions were found at 288.3, 422.4, 434.5, 448.4, 512.4, 538.4, 556.4 and 570.4. It was identified as procyanidin A2 by comparison with reference [11].

The present study found that there was no difference in the individual procyanidin composition of the different litchi varieties. As shown in Table 3, the contents of four procyanidins (procyanidin B2, epicatechin, A-type procyanidin trimer, and procyanidin A2) in all analyzed varieties were in the ranges of 0.13–1.08, 0.74–3.31, 0.17–0.79, and 0.28–1.31 mg EPE/g FW, respectively. Epicatechin and procyanidin A2 are the two main procyanidins of litchi pericarp, which was consistent with the results previously reported by Sarni-Manchado *et al.* [20], and Liu *et al.* [27]. The epicatechin contents in most tested litchi varieties were higher than the level (1.72 mg/g FW) reported by Sarni-Manchado *et al.* [20], but all lower than the level (3.74 mg/g FW) reported by Liu *et al.* [27]. 

Previous studies by Zhao *et al.* [6] on procyanidin profiles of LFP (Huaizhi variety) have suggested that the major flavonoids present were epicatechin, procyanidin B2 and procyanidin B4, while Sarni-Manchado *et al.* [20] identified condensed tannins, epicatechin and procyanidin A2 as the major phenolic compounds in pericarp of Kwai Mi variety. Liu *et al.* [11] confirmed that the procyanidins in pericarp of Kwai Mi variety were epicatechin, A-type procyanidin trimer and procyanidin A2. However, no difference was observed in procyanidin composition of pericarp of all the nine litchi varieties in the present study. These results suggested that procyanidin composition of LFP may be more affected by environmental, post-harvesting conditions and extraction means other than by cultivar.

### 2.4. Total Antioxidant Activity and Relationship to Phytochemical Content

**Figure 3:**
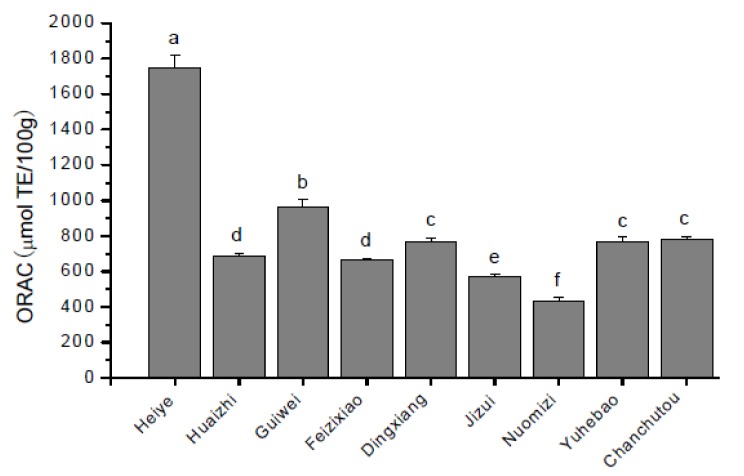
Oxygen radical absorbance capacity (ORAC) of phenolic extracts from the pericarp of nine litchi varieties (mean ± standard deviation, n = 6). Bars with no letters in common are significantly different (*p* < 0.05).

As shown in Figure 3, the ORAC values of the nine litchi varieties ranged from 430.49 to 1752.30 μmol TE/100 g FW, averaging 820.07 μmol TE/100 g FW. The highest ORAC value was observed in Heiye (1752.30 μmol TE/100 g) as expected from its high contents of total phenolics, with a 4.1-fold difference compared to the variety with the lowest ORAC value (Nuomizi, *p* < 0.05).

**Figure 4:**
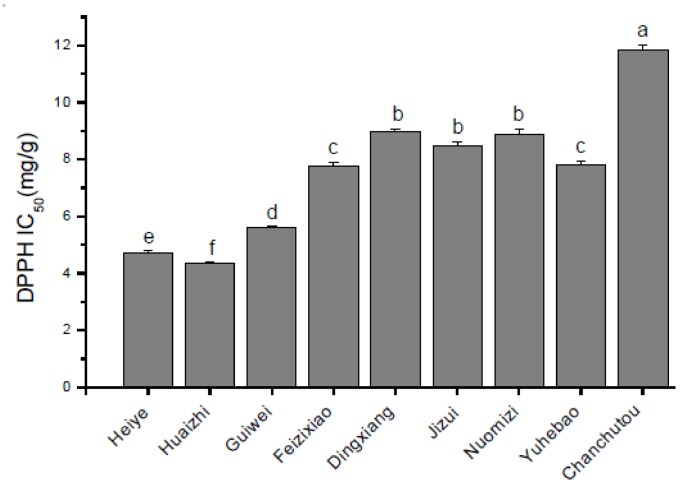
DPPH scavenging activities of phenolic extracts from the pericarp of nine litchi varieties (mean ± standard deviation, n = 6). Bars with no letters in common are significantly different (*p* < 0.05).

The DPPH radical-scavenging activities of litchi pericarp extracts of selected varieties are shown in Figure 4. The average IC_50_ value ranged from 4.70 to 11.82 mg/g. The Huaizhi variety had the most effective DPPH radical scavenging activity, with a 2.7-fold difference observed when compared to the variety with the lowest DPPH radical-scavenging activity (Changchutou, *p* < 0.05).

Grapes and many other fruits have been used as valuable sources of antioxidants. In the present study, Heiye variety showed the strongest antioxidant capacity (1752.30 μmol TE/100 g FW), which is comparable to that of the grape (17.59 μmol TE/g FW), guava (13.06 μmol TE/g FW), pomelo (10.76 μmol TE/g FW) [28]. These valuable properties make LFP a potential source for nutraceutical development.

In the present study, both total phenolic and flavonoid contents were strongly correlated with ORAC activities (r = 0.73, *p* < 0.01; and r = 0.71, *p* < 0.05, respectively). A significant correlation was also found between DPPH activities and total phenolic (r = −0.68, *p* < 0.05) and total flavonoid (r = −0.63, *p* < 0.05) contents. These significant correlations suggested that the phenolics/flavonoids apparently contributed to the antioxidant capacity of LFP. The correlation between total phenolic content and antioxidant activity was also reported in other fruits [29,30], while the correlation of total procyanidins/anthocyanins with ORAC value or DPPH activities did not attain a significant level in this study. This result was different from previous studies in which the antioxidant activity was significantly related to procyanidin and anthocyanin contents [29,30]. More recently, Guendez *et al.* [15] have reported that procyanidin B1 was one of the most important radical scavengers in grape seed extracts, despite its low contribution to overall phenolic content. Hence, the difference may be attributed to different composition and proportion of procyanidins/anthocyanins of the tested litchi varieties.

## 3. Experimental 

### 3.1. Plant Materials

Fresh fruits of nine litchi cultivars at the mature stage were collected from the Institute of Fruit Tree Research, Guangdong Academy of Agricultural Sciences in Guangzhou and Fujian Academy of Agricultural Sciences in Fuzhou, China, in 2008 and 2009. Fresh fruits were peeled after harvest and the pericarp was *kept in sealed polyethylene bags* at −70 °C for further studies. The moisture contents of the LFP samples used in this investigation ranged from 55.5% to 57.0%. The basic information of the nine litchi cultivars is presented in Table 4. 

### 3.2. Chemicals and Reagents

Epicatechin, gallic acid, catechin, cyanidin-3-rutinoside, cyanidin-3-glucoside, malvidin-3-glucoside, 2,2'-azobis(2-amidinopropane) dihydrochloride (AAPH), 6-hydroxy-2,5,7,8-tetramethylchroman-2-carboxylic acid (Trolox), and 3',6'-dihydroxyspiro [isobenzofuran-1(^3^H),9'-(^9^H)xanthene]-3-one, disodium salt (FL) were purchased from Sigma-Aldrich Co. (Shanghai, China). 1,1-Diphenyl-2-picryldydrazyl (DPPH) was purchased from WeiJia Company (Guangzhou, China). HPLC grade methanol, acetonitrile, and formic acid were obtained from Thermo Fisher Scientific Inc. (Shanghai, China). All other chemicals used in this study were of analytical grade and obtained from Sinopharm Chemical Reagent Co. (Shanghai, China).

### 3.3. Extraction of Phenolics

The LFP phenolics were extracted by the method of Wolfe *et al.* [31], with minor modifications. Briefly, LFP (50 g) were homogenized with chilled 80% acetone solution (500 mL) in a Waring blender at high speed for 5 min. The sample was then further homogenized for 3 min using a Polytron homogenizer, and extracted for 2 h at room temperature. The extract was filtered through a membrane (0.45 μm), and the residue was re-extracted and filtered. Filtrates were combined and concentrated under vacuum by a rotary evaporator at 45 °C. The dried extract was dissolved in 50 mL of water and used for further analysis.

### 3.4. Determination of Total Phenolic Content

The total phenolics contents in extracts were estimated using a modified colorimetric Folin-Ciocalteu method [32]. Briefly, LFP extract (0.25 mL) was added to deionized water (1.0 mL) and Folin-Ciocalteu reagent (0.25 mL). After 5 minutes, 7% sodium carbonate solution (2.5 mL) was added, and the mixture was kept for 90 min at room temperature before measurement at 760 nm using a Shimadzu UV-1800 UV-Vis spectrophotometer (Tokyo, Japan). The measurement was compared to a standard curve of gallic acid solutions and expressed as milligrams of gallic acid equivalents (GAE) per gram fresh weight (FW).

### 3.5. Determination of Total Flavonoid Content

The flavonoid contents of LFP were measured using a modified colorimetric method [32,33]. Briefly, extract (0.25 mL) was added to distilled water (1.25 mL) and 5% sodium nitrite solution (75 μL). After standing for 5 min, 10% aluminum chloride (0.15 mL) was added to the solution and allowed to react for 6 min. Then, 1.0 M sodium hydroxide (0.50 mL) was added, and the mixture was diluted with another 0.275 mL of distilled water. The absorbance of the mixture at 510 nm was measured and compared to a standard curve of catechin solution. The flavonoid content was expressed as milligrams of catechin equivalents (CAE) per gram FW.

### 3.6. Determination of Total Anthocyanin Content

Total anthocyanin contents of LFP were measured using a modified spectophotometric pH differential protocol [34]. The absorbance values of LFP extract in 25 mM potassium chloride buffer (pH 1.0) and 0.4 M sodium acetate buffer (pH 4.5) were measured simultaneously at 515 and 700 nm against distilled water blank. The anthocyanin content was calculated as:

Total anthocyanins (mg/100 g of fresh pericarp) = A × MW × 1000/(ε × C)

where A is absorbance = (A_515_ − A_700_)_pH1.0_ − (A_515_ − A_700_)_pH4.5_; MW is molecular weight for cyanidin-3-glucoside = 449.2; ε is the molar absorptivity of cyanidin-3-glucoside = 26900; and C is the concentration of the buffer in mg/mL. The anthocyanin content was expressed as milligrams of cyanidin-3-glucoside equivalents (CGE) per 100 g FW.

### 3.7. Determination of Anthocyanin Composition

The anthocyanin composition and individual content of LFP samples were determined using a HPLC method described by Ju and Howard [35] with some modifications. Briefly, the HPLC was performed on an Xbridge-C18 column (250 × 4.6 mm i.d., 5 μm particle size, Waters, Milford, MA, USA). Solvent A [5% (v/v) aqueous formic acid] and solvent B (methanol) were used as mobile phases. A 10 μL sample solution was injected and the elution conditions were as follows: a linear gradient from 5% to 10% B in 5 min, from 10% to 40% B in 40 min, at a flow rate of 0.9 mL/min. The column was then reequilibrated with 5% B for 10 min before the next injection. The absorbance of the eluate was monitored at 520 nm. The retention time, percentage peak area under the curve, and the spectroscopic data of anthocyanin standards were used to identify the quantity of anthocyanins present in the samples. Spike recovery ranged from 94.6% to 97.5%. The lower detection limit for all anthocyanin standards was <20 ng/mL. The anthocyanin content was expressed as milligrams of anthocyanin per 100 g FW.

### 3.8. Determination of Total Procyanidin Content

Total procyanidin contents of LFP were measured using the method described by Sun *et al.* [36]. Briefly, sample (0.5 mL), 30 g/L aqueous vanillin (2.5 mL) and 30% (v/v) H_2_SO_4_–methanol solution (2.5 mL) were added sequentially. The solution was reacted in the dark for 20 min at 30 °C. The absorbance of the sample was monitored at 500 nm. The measurement was compared to a standard curve of prepared epicatechin solutions and expressed as milligrams of epicatechin equivalents (EPE) per gram FW.

### 3.9. Determination of Procyanidin Composition

Reverse-phase high performance liquid chromatography was used to analyse procyanidins [11], using SB-C18 column (150 × 4.6 mm i.d., 5 μm particle size, Agilent, Santa Clara, CA, USA). Solvent A [0.4% (v/v) aqueous acetic acid] and solvent B (acetonitrile) were used as mobile phases. A 10 μL sample solution was injected and the elution conditions were as follows: a linear gradient from 5% to 15% B in 20 min, from 15% to 25% B in 20 min, from 25% to 35% B in 5 min, from 35% to 50% B in 5 min, and from 50% to 5% B in 5 min, at a flow rate of 1 mL/min. The column was then reequilibrated with 5% B for 5 min before next injection. The absorbance of the eluate was monitored at 280 nm. The spectroscopic data of procyanidin standards (epicatechin) were used to identify the quantity of procyanidins present in the samples. The content of procyanidin was expressed as milligrams epicatechin equivalents (EPE) per gram FW.

A MS system (Esquire HCT PLUS, Bruker, Bremen, Germany), equipped with an electrospray ionization (ESI) interface was used to determine the molecular weight. SB-C18 column (150 × 4.6 mm i.d., 5 μm particle size, Agilent) was used and 10 μL of sample solution were injected into the MS system. The elution conditions were the same as described above, and the effluent was subsequently detected by ESI-MS with a negative ion mode. The orifice voltage was −30 V and a heat capillary temperature of 275 °C. The mass scale was defined from 50 to 1,000 *m/z*.

### 3.10. Determination of Antioxidant Capability

#### 3.10.1. Assay of Oxygen Radical Absorbance Capacity (ORAC)

The ORAC of LFP was measured as previously described [37]. Briefly, 20 μL of blank, Trolox standard, or LFP extracts in 75 mM potassium phosphate buffer, pH 7.4 (working buffer), was added to triplicate wells in a black clear-bottom 96-well microplate. A volume of 200 μL of 0.96 μM FL in working buffer was added to each well and incubated at 37 °C for 20 min, with intermittent shaking. After prompt addition of 20 μL of freshly prepared 119 mM AAPH in working buffer, the microplate was immediately inserted into a Fluoroskan Ascent FL plate reader (Thermo Labsystems, Franklin, MA, USA) at 37 °C. The decay of fluorescence at 538 nm was measured with excitation at 485 nm every 4.5 min for 2.5 h. The areas under the curve (AUC) were calculated by the areas under fluorescence versus time curves for the samples minus the AUC for the blank. The ORAC value of LFP from each variety was obtained by comparison to a standard curve of the AUC for 6.25, 12.5, 25, and 50 μM Trolox standards minus the AUC for blank. ORAC values are expressed as μmol Trolox equivalents (TE)/100 g FW.

#### 3.10.2. DPPH Radical Scavenging Activity

The DPPH radical-scavenging activities of LFP were evaluated by modification of previously reported method [38]. Briefly, 20 μL of each diluted LFP extract of was mixed separately with 90 μM methanolic solution of DPPH radical to a final volume of 1 mL. The disappearance of DPPH radical was monitored by the decrease in absorbance at 515 nm, which was recorded after 0, 1, 2, 3, 4 and 5 min, and subsequently every 5 min up to 120 min, during which time the radical was stable. The scavenging activities were expressed as mg of LFP (FW) required to decrease the initial concentration of DPPH radical by 50% (IC_50_, mg LFP/g DPPH).

### 3.11. Statistical Analysis

Data were expressed as means ± standard deviations and then analysed by SPSS V.13 (SPSS Inc., Chicago, IL, USA). One way analysis of variance (ANOVA) and Duncan’s New Multiple-range test were used to determine the differences among the means. Differences at *p* < 0.05 were considered statistically significant. Relationship between contents of phenolics and antioxidant activity was assessed through correlation and regression.

## 4. Conclusions 

Varietal differences were found in the contents of total phenolics, flavonoids, anthocyanins, procyanidins, and antioxidant activity in litchi pericarp. There were significant differences in anthocyanin compositions and individual contents among the tested litchi varieties. Varietal differences also occurred in the contents of individual procyanidins, while there was no difference in procyanidin compositions. The radical-scavenging activities were related to the levels of total phenolics and flavonoids. This indicated that litchi pericarps rich in phenolics have the potential to be used as functional ingredients for food and pharmaceutical applications.

## Figures and Tables

**Table 1:** Total phenolic, flavonoid, anthocyanin, and procyanidin contents in litchi pericarp of nine varieties.

Variety	Total Phenolics (mg GAE/g FW)	Total Flavonoids (mg CAE/g FW)	Total Anthocyanins (mg CGE/g FW)	Total Procyanidins (mg EPE/g FW)
Heiye	30.16 ± 1.14 ^a^	23.46 ± 1.59 ^a^	18.60 ± 0.14 ^b^	7.46 ± 0.16 ^a^
Huaizhi	25.22 ± 1.09 ^b^	19.19 ± 1.46 ^b^	20.94 ± 0.14 ^a^	6.24 ± 0.15 ^c^
Guiwei	20.30 ± 1.46 ^c^	16.22 ± 1.01 ^c^	5.97 ± 0.13 ^f^	6.55 ± 0.20 ^b^
Feizixiao	14.31 ± 1.19 ^d^	11.90 ± 1.03 ^d^	1.77 ± 0.14 ^i^	3.94 ± 0.17 ^d^
Dingxiang	13.58 ± 0.89 ^de^	10.99 ± 1.35 ^d^	6.64 ± 0.16 ^e^	3.93 ± 0.12 ^d^
Jizui	11.79 ± 1.05 ^ef^	7.12 ± 0.22 ^e^	2.96 ± 0.12 ^h^	1.71 ± 0.13 ^f^
Nuomizi	11.60 ± 1.11 ^eg^	11.35 ± 0.93 ^d^	5.72 ± 0.14 ^g^	6.16 ± 0.24 ^c^
Yuhebao	10.06 ± 1.16 ^fg^	7.49 ± 0.77 ^e^	8.29 ± 0.13 ^c^	3.43 ± 0.18 ^e^
Chanchutou	9.39 ± 0.74 ^g^	7.69 ± 0.60 ^e^	7.75 ± 0.16 ^d^	3.98 ± 0.15 ^d^

Values are expressed as the mean ± standard deviation (n = 6). Means with different superscript letters within a column are significantly different (*p* < 0.05). GAE: gallic acid equivalents, CAE: catechin equivalents, CGE: cyanidin-3-glucoside equivalents EPE: epicatechin equivalents.

**Table 2:** Profiles of anthocyanins in litchi pericarp of nine varieties.

Variety	Cyanidin-3-glucoside (mg/100 g FW)	Cyanidin-3-rutinoside (mg/100 g FW)	Malvidin-3-glucoside (mg/100 g FW)
Heiye	1.18 ± 0.37 ^a^	16.99 ± 1.64 ^b^	1.59 ± 0.27 ^b^
Huaizhi	0.84 ± 0.08 ^b^	19.11 ± 1.43 ^a^	0.78 ± 0.07 ^d^
Guiwei	nd	5.73 ± 0.33 ^d^	1.04 ± 0.09 ^c^
Feizixiao	nd	1.29 ± 0.15 ^g^	nd
Dingxiang	nd	3.80 ± 0.77 ^e^	1.03 ± 0.13 ^c^
Jizui	nd	2.47 ± 0.23 ^f^	0.66 ± 0.02 ^e^
Nuomizi	nd	4.36 ± 0.25 ^e^	1.98 ± 0.17 ^a^
Yuhebao	nd	7.29 ± 0.83 ^c^	0.67 ± 0.05 ^e^
Chanchutou	0.80 ± 0.06 ^b^	6.43 ± 0.44 ^c^	nd

Values are expressed as the mean ± standard deviation (n = 6). Means with different superscript letters within a column are significantly different (*p* < 0.05). nd: not detected.

**Table 3:** Profiles of procyanidins in litchi pericarp of nine varieties.

Variety	Procyanidin B2 (mg EPE/g FW)	Epicatechin (mg EPE/g FW)	A-type Procyanidin trimer (mg EPE/g FW)	Procyanidin A2 (mg EPE/g FW)
Heiye	1.08 ± 0.16 ^a^	3.21 ± 0.07 ^a^	0.79 ± 0.12 ^a^	1.31 ± 0.14 ^a^
Huaizhi	0.56 ± 0.14 ^bc^	3.31 ± 0.05 ^a^	0.61 ± 0.12 ^abc^	1.21 ± 0.19 ^ab^
Guiwei	0.61 ± 0.08 ^bc^	3.28 ± 0.05 ^a^	0.73 ± 0.11 ^ab^	1.21 ± 0.27 ^ab^
Feizixiao	0.29 ± 0.09 ^ef^	1.82 ± 0.13 ^c^	0.29 ± 0.11 ^de^	0.50 ± 0.14 ^c^
Dingxiang	0.43 ± 0.08 ^cde^	1.81 ± 0.04 ^c^	0.43 ± 0.12 ^cd^	0.47 ± 0.09 ^c^
Jizui	0.13 ± 0.07 ^f^	0.74 ± 0.08 ^d^	0.17 ± 0.09 ^e^	0.28 ± 0.08 ^c^
Nuomizi	0.67 ± 0.09 ^b^	2.79 ± 0.05 ^b^	0.54 ± 0.13 ^bc^	1.00 ± 0.21 ^b^
Yuhebao	0.37 ± 0.14 ^de^	1.65 ± 0.12 ^c^	0.31 ± 0.12 ^de^	0.45 ± 0.13 ^c^
Changchutou	0.33 ± 0.06 ^e^	1.63 ± 0.03 ^d^	0.44 ± 0.07 ^cd^	0.57 ± 0.19 ^c^

Values are expressed as the mean ± standard deviation (n = 6). Means with different superscript letters within a column are significantly different (*p* < 0.05). EPE: epicatechin equivalents.

**Table 4:** Description of the nine different litchi cultivars.

Variety	Fruit mass (g)	Pericarp rate (%)	Features of fruit pericarp
Heiye	22.06 ± 2.87 ^d^	15.76 ± 0.15 ^c^	Dark red, smooth
Huaizhi	27.69 ± 2.91 ^c^	19.62 ± 0.10 ^a^	Dark red, smooth
Guiwei	20.05 ± 3.46 ^d^	18.18 ± 1.80 ^a^	Pink and green, sharp protuberances
Feizixiao	27.62 ± 5.21 ^c^	16.61 ± 0.12 ^b^	Pink, sharp protuberances
Dingxiang	39.97 ± 5.76 ^b^	19.69 ± 0.62 ^a^	Purple, tempered protuberances
Jizui	25.01 ± 3.65 ^cd^	18.61 ± 1.44 ^a^	Dark red, tempered protuberances
Nuomizi	26.18 ± 2.47 ^c^	14.68 ± 0.49 ^d^	Cardinal, tempered protuberances
Yuhebao	29.36 ± 2.79 ^c^	15.32 ± 0.36 ^c^	Purple, tempered protuberances
Chanchutou	51.45 ± 5.63 ^a^	20.02 ± 1.14 ^a^	Purple, tempered protuberances

Values are expressed as the mean ± standard deviation (n = 20). Means with different superscript letters within a column are significantly different (*p* < 0.05).
